# A Novel Physical Approach for Cationic–Thiolate Protected Fluorescent Gold Nanoparticles

**DOI:** 10.1038/srep15372

**Published:** 2015-10-20

**Authors:** Yohei Ishida, Chaiyathat Lee, Tetsu Yonezawa

**Affiliations:** 1 Division of Material Science and Engineering, Faculty of Engineering, Hokkaido University, Kita 13, Nishi 8, Kita-ku, Sapporo, Hokkaido 060-8628, Japan

## Abstract

Knowledge on the synthesis of cationically charged fluorescent gold nanoparticles (Au NPs) is limited because the electrostatic repulsion between cationic ligands on the surface of NP hinders the formation of small Au NPs (usually less than *ca*. 2 nm) during nucleation in solvents. We herein propose a novel methodology for a synthesis of water-dispersible, cationic–thiolate protected fluorescent Au NPs by the sputtering of Au into liquid matrix containing thiolate ligands. By controlling mercaptan concentration the size and photophysical characteristics of Au NPs were directly controlled, resulting in near IR fluorescence with a 0.9% of absolute quantum yield. Cationically charged fluorescent metal NPs are promising, especially in biological fields, and this work provides a novel methodology towards the synthesis of a new series of functional metal NPs.

Nanotechnology is one of the most innovative developments of science and engineering in the last several decades. It has been widely investigated in order to fabricate materials with novel properties for various areas of chemical, physical and biological fields^1^,^2^. Interest in inorganic nanoparticles (NPs)^3^,^4^ such as metal NPs and quantum dots as optical materials is increasing because of their unique optical and electronic properties^5^,^6^,^7^. In particular, gold nanoparticles (Au NPs) have been studied intensively because of their chemical stability and nontoxicity^8^,^9^,^10^,^11^. For noble metal NPs comprising Au NPs, the property of surface plasmon absorption or fluorescence along with their quantum size effects has encouraged their application as color pigments or bio-sensing materials^12^,^13^. For these applications, size control of Au NPs is the most important, and many molecules such as amines, mercaptans, or polymers have been used as capping reagents because of their good coordination properties with gold^5^,^9^,^11^. With respect to fluorescence properties, the size of Au NPs directly affects their band-gap^12^,^13^,^14^, and thus can control peak positions and quantum yields. A typical approach for well-ordered Au NPs is a chemical reduction of Au ions in a solution with a reducing agent such as NaBH_4_^7^,^9^,^11^, hydrazine, alcohol^4^, polyol, or citric acid^5^. These methods have been extensively developed, however the contamination and formation of by-products and impurities is a disadvantage. There are only a few reports on environmentally–friendly methods for metal NP synthesis and their use^15^,^16^,^17^. Because complicated procedures are involved in the purification and separation of products, simple processes for preparing NPs are required.

Recently, metal sputtering over liquid matrix^18^,^19^,^20^,^21^,^22^,^23^,^24^,^25^,^26^ has been demonstrated in order to prepare colloidal metal NPs without the use of reductants. Moreover, we have developed this approach for organic–protected metal NPs, and termed it as the “matrix sputtering method”^18^,^19^,^20^,^21^,^22^,^23^. In a sputtering process, argon or nitrogen is ionized by a high voltage, which then attacks the target metal. Ejected atoms or clusters coalesce not only in the gas phase but also inside or on the interface of the liquid matrix and form NPs. Several liquid matrixes have been used for the preparation of Au NPs using this method, and they can be categorized by their functional groups into (a) with mercapto groups such as 6-mercaptohexyltrimethylammoniumbromide^18^, pentaerythritol tetrakis (3-mercaptopropionate)^19^, and (b) without mercapto groups such as polyethylene glycol^20^,^21^,^22^, ionic liquids^25^,^26^, and pentaerythritol ethoxylate^19^. Liquid matrixes in group (a) resulted in very small (~1.3 nm) fluorescent Au NPs, while those in group (b) formed rather large Au NPs (2–5 nm). The difference in their size was attributed to the coordination property of mercapto groups in group (a) that can prevent the coalescence of Au NPs inside or on the interface of liquid matrices. Moreover, this technique can be applied for various conductive materials such as Ag^22^ and Cu^23^. From these results, it is hypothesized that we can prepare metal NPs of various sizes of the order of a single nanometer, and thus, can control the physical properties of NPs by matrix sputtering method.

In this paper, we present a first synthesis of cationically charged fluorescent Au NPs with precisely controlled diameters using the matrix sputtering method. While previous approaches have only used liquid matrices, our new approach uses both a liquid matrix and a solid organic molecule with mercapto groups. The organic molecule cannot be used as the matrix because it is in the solid form at room temperature with non–melting property (it decomposes at 173 °C without melting, see TG–DTA spectra shown in Fig. S1). The stabilizing molecules can dissolve in a liquid matrix and prevent the coalescence of Au NPs inside or on the interface of the liquid matrix, and their concentration successfully controlled the diameter of Au NPs of the order of a single nanometer. To the best of our knowledge, little is known about the synthesis of water-dispersible fluorescent Au NPs using cationic mercaptan ligands except for only a few reports^18^, because the electrostatic repulsion between cationic ligands on the surface of NP hinders the formation of small Au NPs (less than 2 nm) during nucleation in solvents. Cationically charged fluorescent Au NPs are promising especially in biological fields, for example, in bio-electronic devices and in targeted drug delivery^27^,^28^. The method presented here will provide a novel methodology for the synthesis of a new series of functional metal NPs.

## Results and Discussion

**Figure 1:**
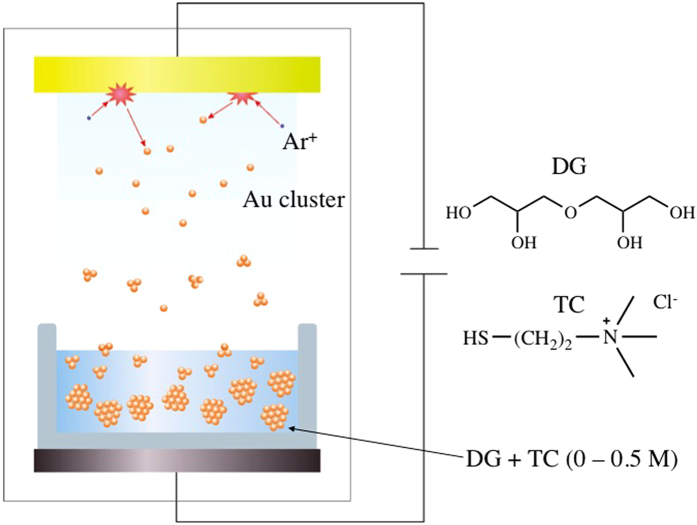
Schematic illustration of the matrix sputtering method and chemical structure of diglycerol (DG) and thiocholine chloride (TC).

The liquid matrix used in this work was diglycerol (DG, b.p. = 412 °C at 760 mmHg) in order to dissolve hydrophilic ligands for this study. The cationic stabilizer molecule, thiocholine chloride (TC, Fig. 1), was synthesized by the hydrolysis of commercially available acetylthiocholine iodide and then the counter ion was exchanged to Cl^−^ according to a method described in a previous paper^29^,^30^. TC is solid at room temperature, and using TG–DTA analysis decomposition was observed at 173 °C instead of the observation of a melting point (Fig. S1). Thus, the use of liquid matrix (DG) is required for the sputtering synthesis of TC-stabilized Au NPs. The experimental setup for the sputtering synthesis is illustrated in Fig. 1. The concentration of TC in DG was set at 0, 0.01, 0.1, and 0.5 M, and TC completely dissolved in DG even at 0.5 M concentration. The thiol group of TC has a good affinity to Au due to the formation of a coordination bond between Au^0^ and the lone pair of electrons of sulfur, or a covalent bond of Au–SR. It is expected that the steric hindrance by the coordination of TC suppresses the growth of Au NPs during the sputtering deposition. Judging from TC’s salt molecular structure, it is expected that TC has almost no vapor pressure and thus the stabilization for nanoparticles should be a liquid process. This mechanism is rather different from our previous case, where α–thioglycerol ligand can coordinate to nanoparticles in a gas phase due to its relatively low boiling point^20^. It should be noted that Au thin film did not form on the liquid matrix, while it is usually deposited on solid surfaces for the purpose of SEM measurement.

**Figure 2:**
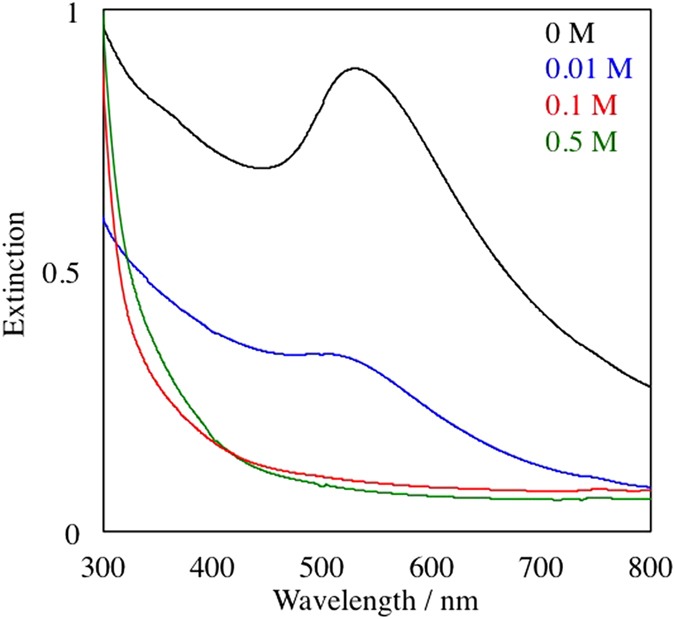
Extinction spectra of Au NPs prepared at various concentration of TC from 0 to 0.5 M in DG. The spectra were measured in a 1 mm quartz cell without purification.

After the sputtering of Au into the matrix, extinction spectra of Au NPs suspension in DG were measured in a quartz cell with 1 mm optical path just after the sputtering preparation without further purification or dilution (Fig. 2). Broad absorption peaks around 520 nm, which are characterized as the localized plasmon absorption of Au NPs, were observed for 0 M and 0.01 M samples. On the other hand, the absorption spectra did not show any plasmon absorption peak for the 0.1 M and 0.5 M samples. Fundamentally, surface plasmon absorption originates from the vibration of free electrons on the surface of NPs resulting in an absorption peak corresponding to the vibration frequencies. Therefore, the generation of plasmon absorption needs a certain particle size (over *ca.* 2.4 nm for Au NPs, as reported in a previous paper^31^). As the number of atoms in each particle decreases, the energy band gap becomes wider according to the quantum size effect. Then, plasmon absorption is not observable in such small particles. Judging from the extinction spectra in Fig. 2, the size of Au NPs would decrease with an increase in the TC concentration, and those prepared with 0.1 M and 0.5 M TC should be very small, and thus, no plasmon absorption was observed. The obtained Au NPs prepared in 0.1 M and 0.5 M TC were water-soluble, as judged by the addition of 3 mL of water into 300 μL of DG dispersion.

**Figure 3:**
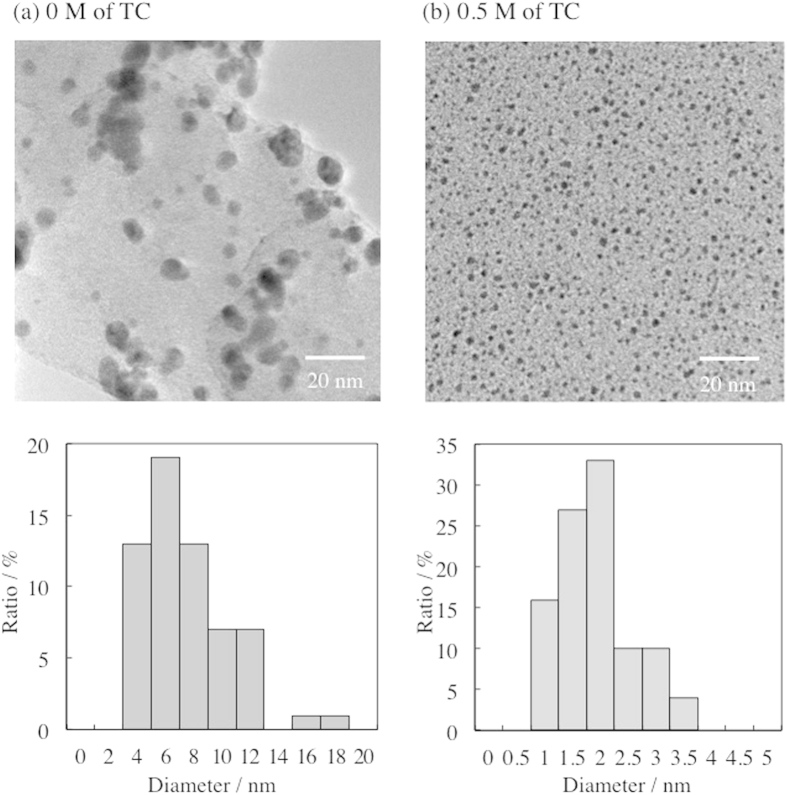
TEM images and size-distribution histograms of Au NPs prepared at 0 and 0.5 M concentration of TC in DG. For the histograms, total 200 particles from more than 5 TEM images were counted.

**Figure 4:**
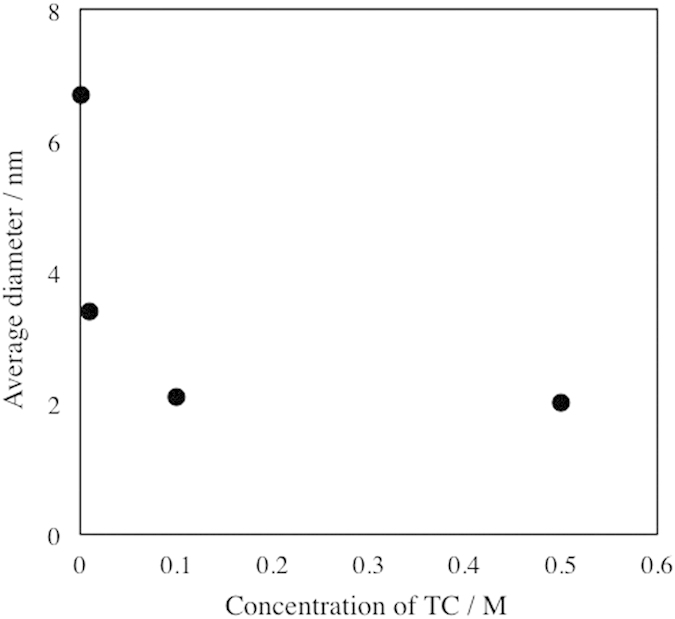
Size of Au NPs prepared at various concentrations of TC from 0 to 0.5 M in DG.

Figure 3 shows the representative transmission electron microscopy (TEM) images and size-distribution histograms of Au NPs prepared at 0 and 0.5 M TC. The average sizes of Au NPs prepared at 0 and 0.5 M of TC were 6.7 ± 3.2 and 2.0 ± 0.7 nm, respectively. This difference in their particle sizes reflects the change in plasmon absorption, as shown in Fig. 2. The change in particle size as a function of TC concentration is shown in Fig. 4. From Fig. 4, it is obvious that a higher TC concentration produces smaller Au NPs. This tendency can be simply explained by the collision probability between TC and Au NPs inside and at the interface of DG. Thus, this result clearly indicates that the size of Au NPs is controllable by the concentration of the thiol-stabilizer in the liquid matrix.

**Figure 5:**
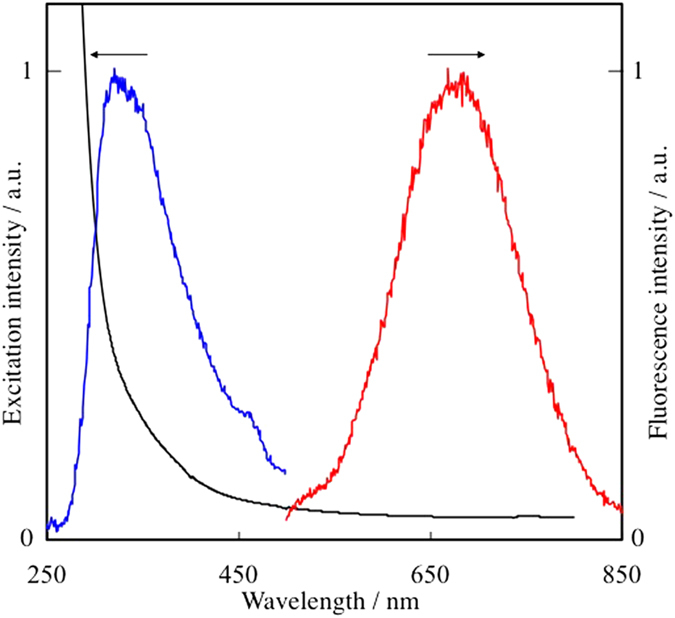
Excitation (blue) and fluorescence (red) spectra of Au NPs prepared at 0.5 M TC in DG. Excitation spectrum was observed at 673 nm. Fluorescence spectrum was observed by the irradiation at 300 nm. For a comparison purpose, the extinction spectrum under the same condition is shown by a black line.

The absence of plasmon absorption and very small particle sizes led us to investigate the fluorescence property of Au NPs stabilized by TC. The excitation wavelength was set at 300 nm. Au NPs prepared at 0 and 0.01 M TC, which show plasmon absorption in Fig. 2, did not fluoresce. On the other hand, those prepared at 0.1 and 0.5 M TC showed fluorescence in the near IR region, as shown by the red line in Fig. 5 (only for the 0.5 M sample). The fluorescence spectra were almost the same for the 0.1 and 0.5 M samples, resulting from the similar average sizes of the Au NPs (2.1 and 2.0 nm for 0.1 and 0.5 M samples, respectively), within errors. The excitation spectrum of the 0.5 M sample was observed at the fluorescence maximum (673 nm) as shown in the blue line in Fig. 5. The excitation maximum was observed at 326 nm; therefore, the Stokes shift of our Au NPs can be calculated to be 347 nm (1.96 eV = 1.58 × 10^4^ cm^−1^). The large Stokes shift of Au NPs has been reported in our previous matrix sputtering synthesis^18^, and the reason was assumed to be the stabilization of the d band and/or the destabilization of the sp–conduction band in the Au NPs that usually results in a blue shift in the absorption (excitation) spectrum.

The diameter of current TC stabilized Au NPs is considerably larger than usual fluorescent Au nanoclusters synthesized by chemical-reduction method^32^,^33^,^34^,^35^. Typically, Au NPs with *ca.* 2 nm of diameter do not fluoresce by a conventional chemical reduction method. Only the very small Au clusters such as Au_8_, Au_25_ *etc*. fluoresce in different wavelength regions according to their core sizes. However, TC stabilized Au NPs with a 2 nm of diameter showed near IR fluorescence with a relatively large Stokes shift. This diameter is significantly larger than that reported previously for fluorescence Au clusters. By a comparison with known Au clusters prepared by a chemical reduction method, the photophysical characteristics of our TC stabilized Au NPs are similar to those of a Au_6_ complex (excitation spectrum from 300 to 420 nm and fluorescence spectrum from 600 to 850 nm) reported in literature^34^. It should be noted that the polarity of thiolate ligands sometimes affects the fluorescence property of Au clusters^35^.

The fluorescence quantum yield of the synthesized Au NPs prepared at 0.1 and 0.5 M TC was measured using the absolute method. Both samples showed 0.9% quantum yield, and this value is similar to those of gold nanoparticles and nanoclusters prepared by various synthetic methods including sputtering and chemical reduction methods^32^,^33^. These photophysical characteristics (fluorescence and excitation wavelengths, fluorescence quantum yield) remained even when the solution was diluted in water by 10 times (v/v).

In conclusion, we presented a novel synthesis of water-dispersible, cationic thiolate-stabilized fluorescent Au NPs with precisely controlled diameters by the sputtering of Au in DG containing TC. While only liquid matrix has been used in previous sputtering methods, both liquid matrix (DG) and organic molecules with mercapto groups (TC) are used in our new approach. The size and photophysical characteristics of Au NPs could be directly controlled by controlling the mercaptan concentration, resulting in a near IR fluorescence with 0.9% quantum yield. Cationically charged fluorescent Au NPs are promising, especially in biological fields^27^,^28^; therefore, this work will provide a novel methodology for the synthesis of a new series of cationic, functional metal NPs.

## Methods

Diglycerol and acetylthiocholine iodide were obtained from Aldrich. Thiocholine chloride was synthesized by the hydrolysis of acetylthiocholine iodide and then the counter ion was exchanged to Cl^−^ (Organo Amberlite IRA400JCL) according to a method described in a previous paper^28^,^29^. The purity of TC was confirmed by FT-IR and ^1^H-NMR. Au target (99.9%) was supplied by Tanaka Precious Metals (Japan).

The experimental procedure for sputtering synthesis is described below. Firstly, to remove volatile substances, DG and TC were dried under vacuum at 100 °C for 2 h under stirring. DG (12.8 g = 10 mL) and the corresponding amount of TC was put in a glass petri dish with a diameter of 6.5 cm, and was horizontally positioned against the sputtering target. The concentration of TC in DG was set at 0, 0.01, 0.1, and 0.5 M (corresponding to 0, 15.6, 156, and 779 mg of TC, respectively), and TC completely dissolved in DG even at 0.5 M concentration. Au was sputtered using a current of 30 mA under Ar at a pressure of 20 Pa. The distance between the surface of DG and the surface of the gold target was 25 mm. Sputtering was carried out for 30 min at room temperature under stirring at 100 rpm.

## Additional Information

**How to cite this article**: Ishida, Y. *et al.* A Novel Physical Approach for Cationic-Thiolate Protected Fluorescent Gold Nanoparticles. *Sci. Rep.* **5**, 15372; doi: 10.1038/srep15372 (2015).

## Supplementary Material

Supplementary Information

## References

[b1] Chen G., Qiu H., Prasad P. N. & Chen X. Upconversion Nanoparticles: Design, Nanochemistry, and Applications in Theranostics. Chem. Rev. 114, 5161–5214 (2014). 24605868 10.1021/cr400425h PMC4039352

[b2] Ariga K. *et al.* Layer-by-Layer Nanoarchitectonics: Invention, Innovation, and Evolution. Chem. Lett. 43, 36–68 (2014).

[b3] Schmid G. Ed. Clusters and Colloids: From Theory to Applications. Wiely-VCH, Weinheim (1994).

[b4] Toshima N. & Yonezawa T. Bimetallic nanoparticles – novel materials for chemical and physical applications. New J. Chem. 22, 1179–1201 (1998).

[b5] Talapin D. V., Lee J.-S., Kovalenko M. V. & Shevchenko E. V. Prospects of Colloidal Nanocrystals for Electronic and Optoelectronic Applications. Chem. Rev. 110, 389–458 (2010). 19958036 10.1021/cr900137k

[b6] Redl F. X., Cho K.-S., Murray C. B. & O’Brien S. Three-Dimensional Binary Superlattices of Magnetic Nanocrystals and Semiconductor Quantum Dots. Nature 423, 968–971 (2003). 12827196 10.1038/nature01702

[b7] Kiely C. *et al.* J. Spontaneous Ordering of Bimodal Ensembles of Nanoscopic Gold Clusters. Nature 396, 444–446 (1998).

[b8] Dreaden E. C. *et al.* The Golden Age: Gold Nanoparticles for Biomedicine. Chem. Soc. Rev. 41, 2740–2779 (2012). 22109657 10.1039/c1cs15237h PMC5876014

[b9] Tsukuda T. Toward an Atomic-Level Understanding of Size-Specific Properties of Protected and Stabilized Gold Clusters. Bull. Chem. Soc. Jpn. 85, 151–168 (2012).

[b10] Taketoshi A. & Haruta M. Size- and Structure-Specificity in Catalysis by Gold Clusters. Chem. Lett. 43, 380–387 (2014).

[b11] Yamanoi Y. *et al.* Chem. Eur. J. 12, 314–323 (2006).16208724

[b12] Wu Z. & Jin R. On the Ligand’s Role in the Fluorescence of Gold Nanoclusters. Nano Lett. 10, 2568–2573 (2010). 20550101 10.1021/nl101225f

[b13] Arguinzoniz A. G. *et al.* Light Harvesting and Photoemission by Nanoparticles for Photodynamic Therapy. Part. Part. Syst. Charact. 31, 46–75 (2013).

[b14] Schaeffer N. *et al.* Fluorescent or Not ? Size-Dependent Fluorescence Switching for Polymer-Stabilized Gold Clusters in the 1.1–1.7 Nm Size Range. Chem. Commun. 34, 3986–3988 (2008). 10.1039/b809876j 18758601

[b15] Naik R. R. *et al.* Biomimetic Synthesis and Patterning of Silver Nanoparticles. Nat. Mater. 1, 169–172 (2002). 12618805 10.1038/nmat758

[b16] Raveendran P., Fu J. & Wallen S. L. Completely “Green” Synthesis and Stabilization of Metal Nanoparticles. J. Am. Chem. Soc. 125, 13940–13941 (2003). 14611213 10.1021/ja029267j

[b17] Kumar A., Vemula P. K., Ajayan P. M. & John G. Silver-Nanoparticle-Embedded Antimicrobial Paints Based on Vegetable Oil. Nat. Mater. 7, 236–241 (2008). 18204453 10.1038/nmat2099

[b18] Shishino Y., Yonezawa T., Kawai K. & Nishihara H. Molten Matrix Sputtering Synthesis of Water-Soluble Luminescent Au Nanoparticles with a Large Stokes Shift. Chem. Commun. 46, 7211–7213 (2010). 10.1039/c0cc01702g 20740224

[b19] Shishino Y. *et al.* Preparation of Optical Resins Containing Dispersed Gold Nanoparticles by the Matrix Sputtering Method. Angew. Chem. Int. Ed. 50, 703–705 (2010). 10.1002/anie.201005723 21226158

[b20] Sumi T. *et al.* Formation and Optical Properties of Fluorescent Gold Nanoparticles Obtained by Matrix Sputtering Method with Volatile Mercaptan Molecules in the Vacuum Chamber and Consideration of Their Structures. Langmuir 31, 4323–4329 (2015). 25773272 10.1021/acs.langmuir.5b00294

[b21] Ishida Y., Sumi T. & Yonezawa T. Sputtering synthesis and optical investigation of octadecanethiol-protected fluorescent Au nanoparticles. New J. Chem. 39, 5895–5897 (2015).

[b22] Ishida Y., Nakabayashi R., Matsubara M. & Yonezawa T. Silver sputtering into a liquid matrix containing mercaptans: the systematic size control of silver nanoparticles in single nanometer-orders. New J. Chem. 39, 4227–4230 (2015).

[b23] Nakagawa K., Narushima T., Udagawa S. & Yonezawa T. Preparation of Copper Nanoparticles in Liquid by Matrix Sputtering Process. J. Phys. Conf. Ser. 417, 012038 (2013).

[b24] Hatakeyama Y. *et al.* Synthesis of Gold Nanoparticles in Liquid Polyethylene Glycol by Sputter Deposition and Temperature Effects on Their Size and Shape. J. Phys. Chem. C 115, 3279–3285 (2011).

[b25] Torimoto T. *et al.* Sputter Deposition Onto Ionic Liquids: Simple and Clean Synthesis of Highly Dispersed Ultrafine Metal Nanoparticles. Appl. Phys. Lett. 89, 243117 (2006).

[b26] Castro H. P. S. *et al.* Third-Order Nonlinear Optical Response of Colloidal Gold Nanoparticles Prepared by Sputtering Deposition. J. Appl. Phys. 114, 183104 (2013).

[b27] Murphy C. J. *et al.* Gold Nanoparticles in Biology: Beyond Toxicity to Cellular Imaging. Acc. Chem. Res. 41, 1721–1730 (2008). 18712884 10.1021/ar800035u

[b28] Sperling R. A. *et al.* Biological Applications of Gold Nanoparticles. Chem. Soc. Rev. 37, 1896–1908 (2008). 18762838 10.1039/b712170a

[b29] Yonezawa T., Genda H. & Koumoto K. Cationic Silver Nanoparticles Dispersed in Water Prepared From Insoluble Salts. Chem. Lett. 32, 194–195 (2003).

[b30] Ishida Y., Jirasupangkul T. & Yonezawa T. One-pot preparation of cationic charged Pt nanoparticles by the autocatalytic hydrolysis of acetylthiocholine. New J. Chem. 39, 4214–4217 (2015).

[b31] Kumara C., Zuo X., Cullen D. A. & Dass A. Faradaurate-940: Synthesis, Mass Spectrometry, Electron Microscopy, High-Energy X-Ray Diffraction, and X-Ray Scattering Study of Au ~940 ± 20(SR) ~160 ± 4 Nanocrystals. ACS Nano 8, 6431–6439 (2014). 24813022 10.1021/nn501970v

[b32] Yu P., Wen X., Toh Y.-R. & Tang J. Temperature-Dependent Fluorescence in Au10 Nanoclusters. J. Phys. Chem. C 116, 6567–6571 (2012).

[b33] Chen T. *et al.* Gold Nanocluster-Conjugated Amphiphilic Block Copolymer for Tumor-Targeted Drug Delivery. ACS Appl. Mater. Interfaces 4, 5766–5774 (2012). 23043448 10.1021/am301223n

[b34] Yam V. W.-W., Cheng E. C.-C. & Cheung K.-K. A Novel High- Nuclearity Luminescent Gold (I)-Sulfido Complex. Angew. Chem., Int. Ed. 38, 197−199 (1999).

[b35] Wang G. *et al.* NIR Luminescence Intensities Increase Linearly with Proportion of Polar Thiolate Ligands in Protecting Monolayers of Au38 and Au140 Quantum Dots. J. Phys. Chem. B 110, 20282−20289 (2006). 17034208 10.1021/jp0640528

