# Supplementary material for: A Novel Physical Approach for Cationic–Thiolate Protected Fluorescent Gold Nanoparticles
Source: Sci Rep. 2015 Oct 20;5:15372. doi: 10.1038/srep15372 (PMC4612736; doi:10.1038/srep15372)
Supplement: Supplementary Information [file srep15372-s1.doc]

**Supporting Information for**

A Novel Physical Approach for Cationic–Thiolate Protected Fluorescent Gold Nanoparticles

Yohei Ishida, Chaiyathat Lee,^†^ and Tetsu Yonezawa*

Division of Material Science and Engineering, Faculty of Engineering, Hokkaido University, Kita 13, Nishi 8, Kita-ku, Sapporo, Hokkaido 060-8628, Japan.

^†^Undergraduate Student, International School of Engineering, Chulalongkorn University, 254 Phyathai Road, Patumwan, Bangkok 10330, Thailand.

*tetsu@eng.hokudai.ac.jp

**Experimental details**

UV-Vis extinction spectra were measured using a spectral photometer (JASCO, V–630, 300 - 800 nm) with a quartz cell of 1 mm optical path, immediately after the sputtering preparation without dilution and purification.

Fluorescence spectra were measured using a fluorescence spectrophotometer (JASCO, FP-6600) with a quartz cell of 10 mm optical path. The filter (Sharp cut filter Y-50) which cut the emission less than 500 nm, was equipped in front of the detection window. Fluorescence quantum yields were recorded with a florescence spectrophotometer (JASCO, FP-6600) equipped with a JASCO ILF-533 integrating sphere unit. Sample was set with a quartz sample cell of 10 mm optical path. The measurement was carried out with a fixed excitation wavelength of each sample. The fluorescent wavelength was measured in the range of 200~950 nm. Au nanoparticle dispersion was used after diluted 5-fold with pure DG.

TEM observation for the size and shape of Au NPs was carried out using JEOL 2010F (acceleration voltage of 200 kV). TEM samples were prepared by dropping gold dispersion of DG onto collodion-coated copper grids. The grids were then soaked into methanol for 30 min in order to remove the excess DG and dried under vacuum.


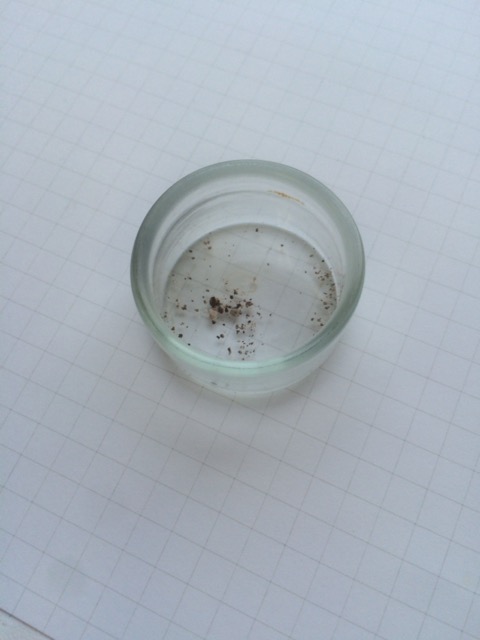


Figure S1. Left: TG–DTA analysis for thiocholine chloride (TC) under N_2_ flow (200 mL / min) at 5 ºC / min of temperature increasing rate. A decomposition temperature was observed at 173 ºC and a melting point was not observed. Right: Sample image of TC heated up to 200 ºC under air. It turned black due to burn without melt.
